# Absence of an embryonic stem cell DNA methylation signature in human cancer

**DOI:** 10.1186/s12885-019-5932-6

**Published:** 2019-07-19

**Authors:** Ze Zhang, John K. Wiencke, Devin C. Koestler, Lucas A. Salas, Brock C. Christensen, Karl T. Kelsey

**Affiliations:** 1 Department of Epidemiology, School of Public Health, Brown University, Providence, RI USA; 2 Department of Neurological Surgery, Institute for Human Genetics, University of California San Francisco, San Francisco, CA USA; 3 Department of Biostatistics, University of Kansas Medical Center, Kansas City, KS USA; 4 Department of Epidemiology, Geisel School of Medicine, Dartmouth College, Lebanon, NH USA; 5 Departments of Molecular and Systems Biology, and Community and Family Medicine, Geisel School of Medicine, Dartmouth College, Lebanon, NH USA; 6 Department of Pathology and Laboratory Medicine, Brown University, Providence, RI USA

**Keywords:** Human embryonic stem cells, Cell differentiation, DNA methylation, Cancer Epigenomics, Biomarkers

## Abstract

**Background:**

Differentiated cells that arise from stem cells in early development contain DNA methylation features that provide a memory trace of their fetal cell origin (FCO). The FCO signature was developed to estimate the proportion of cells in a mixture of cell types that are of fetal origin and are reminiscent of embryonic stem cell lineage. Here we implemented the FCO signature estimation method to compare the fraction of cells with the FCO signature in tumor tissues and their corresponding nontumor normal tissues.

**Methods:**

We applied our FCO algorithm to discovery data sets obtained from The Cancer Genome Atlas (TCGA) and replication data sets obtained from the Gene Expression Omnibus (GEO) data repository. Wilcoxon rank sum tests, linear regression models with adjustments for potential confounders and non-parametric randomization-based tests were used to test the association of FCO proportion between tumor tissues and nontumor normal tissues. *P*-values of < 0.05 were considered statistically significant.

**Results:**

Across 20 different tumor types we observed a consistently lower FCO signature in tumor tissues compared with nontumor normal tissues, with 18 observed to have significantly lower FCO fractions in tumor tissue (total *n* = 6,795 tumor, *n* = 922 nontumor, *P* < 0.05). We replicated our findings in 15 tumor types using data from independent subjects in 15 publicly available data sets (total *n* = 740 tumor, *n* = 424 nontumor, *P* < 0.05).

**Conclusions:**

The results suggest that cancer development itself is substantially devoid of recapitulation of normal embryologic processes. Our results emphasize the distinction between DNA methylation in normal tightly regulated stem cell driven differentiation and cancer stem cell reprogramming that involves altered methylation in the service of great cell heterogeneity and plasticity.

**Electronic supplementary material:**

The online version of this article (10.1186/s12885-019-5932-6) contains supplementary material, which is available to authorized users.

## Background

Many cancerous tumors have long been known to acquire histologic characteristics devoid of the defining features of the tissue of origin. This process of dedifferentiation is characterized by cell regression from a specialized function to a simpler state reminiscent of stem cells [1]. The dedifferentiation of normal cells has long been one theory of the cellular origin of cancers, with the process of dedifferentiation posited to give rise to cancer stem cells; an alternative suggests that cancer stem cells arise from adult stem cells present in the tissues [2]. These cancer stem cells, then, have been suggested to be a subpopulation of malignant cells similar to normal stem cells, having many characteristics of stemness, including self-renewal, differentiation, and proliferative potential [3]. They have been posited to be responsible for genesis of all of the tumor cells in a malignancy and thus been known as “tumor-initiating cells” or “tumorigenic cells” [4, 5]. Putative cancer stem cells have been identified in a number of solid tumors, including breast cancer [6], brain tumors [7], lung cancer [8], colon cancer [9], and melanoma [10]. Studies have shown that cancer stem cells play a crucial role in the genesis of resistance to chemotherapeutic agents, suggesting that these cells may be responsible for disease recurrence [11, 12]. Cancer stem cells are also implicated in serving as the basis of metastases [13, 14].

Studies focusing on somatic cell reprogramming have underscored the similarity between cancer stem cells and induced pluripotent stem cells [15, 16], and the acquisition of pluripotency during the reprogramming process is reminiscent of the dedifferentiation long observed during the process of carcinogenesis [17]. Moreover, studies have shown that cancer stem cells and embryonic stem cells (ESC) have similar cell surface markers [18, 19]. It has been hypothesized that the similarities shared by cancer stem cells and embryonic stem cells might relate to their shared patterns of gene expression and gene regulation [20]. In an effort to account for the self-renewing properties of cancer stem cells, several investigators have defined ‘embryonic stem cell specific expression’ signatures, and these have been analyzed and found in multiple cancers [21–23]. Cancer stem cells exhibit ESC-like signatures that include activation of the oncogene *c-MYC* and similar alterations to important loci responsible for the genesis of pluripotency such as: *SOX2, DNMT1, CBX3 and HDAC1* [19, 20]. Programming the cancer stem cell phenotypes are genetic alterations and epigenetic changes in chromatin structure and DNA methylation [24, 25]. The consequence of cancer stem cell epigenetic alterations is to unleash cellular plasticity that favors oncogenic cellular reprogramming [26].

During normal development stem cell maturation can be traced using DNA methylation. Recently, we devised the fetal cell origin (FCO) DNA methylation signature to estimate fractions of cells that are of fetal origin using 27 ontogeny informative CpG loci [27]. The fetal origin cells are defined as cells that are differentiated from fetal stem cells as compared to adult stem cells. Using a fetal cell reference methylation library and a constrained quadratic programming algorithm, we demonstrated a high proportion of cells with the FCO signature in diverse fetal tissue types and, in sharp contrast, minimal proportions of cells with the FCO signature in corresponding adult tissues [27]. The FCO signature is highly reminiscent of embryonic stem cell lineage and is observed in high levels among embryonic stem cell lines, induced pluripotent stem cells, and fetal progenitor cells [27]. The FCO signature represents a stable phenotypic block of CpG sites that are transmitted from stem cell progenitors to progeny cells across lineages. As such the FCO is a mark of epigenome stability in differentiating tissues. Here, we implemented the FCO signature to infer and then compare the fetal cell origin fractions in thousands of tumor tissues, comprising different cancer types, as well as corresponding nontumor normal tissues. Given the longstanding hypothesis that dedifferentiation in the development of malignancies involves the generation of cancer stem cells, along with the similarities between embryonic stem cells and tumor cells, we hypothesized that the fetal cell origin signal in tumor tissue would be increased compared to nontumor normal tissue.

## Methods

### Discovery data sets

Level 3 Illumina Infinium HumanMethylation450 BeadChip array data collected on tumor tissues and nontumor normal tissues from 21 TCGA studies were considered in our analysis. This included: bladder urothelial carcinoma (BLCA), breast invasive carcinoma (BRCA), cervical squamous cell carcinoma and endocervical adenocarcinoma (CESC), cholangiocarcinoma (CHOL), colon adenocarcinoma (COAD), esophageal carcinoma (ESCA), glioblastoma multiforme (GBM), head and neck squamous cell carcinoma (HNSC), kidney renal clear cell carcinoma (KIRC), liver hepatocellular carcinoma (LIHC), pheochromocytoma and paraganglioma (PCPG), lung adenocarcinoma (LUAD), lung squamous cell carcinoma (LUSC), pancreatic adenocarcinoma (PAAD), prostate adenocarcinoma (PRAD), rectum adenocarcinoma (READ), sarcoma (SARC), stomach adenocarcinoma (STAD), thyroid carcinoma (THCA), thymoma (THYM) and uterine corpus endometrial carcinoma (UCEC). Among the 21 candidate TCGA studies, five: THYM, PCPG, CESC, GBM and STAD, had fewer than 3 nontumor normal samples with available DNA methylation data. To increase the number of samples with methylation profiles in nontumor normal tissue for the five previously mentioned studies we scanned the Gene Expression Omnibus (GEO) data repository to locate data sets we could draw on to enrich the numbers of nontumor normal samples. We were able to add nontumor normal samples of cervix, brain, adrenal gland and stomach from GEO data sets GSE46306 [28], GSE80970 [29], GSE77871 [30] and GSE103186 [31] to cervical squamous cell carcinoma and endocervical adenocarcinoma, glioblastoma multiforme, pheochromocytoma and stomach adenocarcinoma projects on TCGA. As we were unable to find additional nontumor normal samples with DNA methylation profiling of the thymus, the thymoma data set was excluded from our final analysis. In total, 20 TCGA studies, including DNA methylation profiling of 6,795 primary tumor tissue samples and 922 nontumor normal tissue samples were included in our analysis.

### Comparison of predicted FCO between tumor tissue and nontumor normal tissue

We first estimated the FCO based on the DNA methylation signatures for each of the 6,795 primary tumor tissue samples and 922 nontumor normal tissue samples. FCO was estimated based on a previously described procedure [27] using 25 of the 27 CpGs comprising the FCO library because two probes were removed in TCGA methylation data due to quality control. A Wilcoxon rank sum test was fit independently to each TCGA study and used to compare the predicted FCO in tumor versus nontumor normal tissue. As patient-level clinical/demographic characteristics could confound the association between the predicted FCO and tumor/nontumor status, we also fit a series of linear regression models to examine the association between predicted FCO and tumor/nontumor status adjusting for potential confounders. Linear regression models were fit independently to each TCGA study and modeled predicted FCO as the response against tumor/nontumor status, with adjustment for age, gender, race and vital status, provided these data were available and relevant to adjust for. All four of the previously mentioned variables were adjusted for in linear regression models fit to the BLCA, BRCA, CHOL, COAD, ESCA, HNSC, KIRC, LIHC, LUAD, LUSC, PAAD, SARC, READ and THCA data sets. As all samples in the UCEC came from female subjects, only age, race and vital status were adjusted for in the analysis of this data set. For READ, only age, gender and vital status were adjusted for due to the lack of race information. For GBM only age and gender were adjusted for due to the lack of information on race and vital status. As a large number of patients in the STAD, PCPG and CESC studies were missing information on gender, race, age and vital status, unadjusted linear regression models were fit to these studies. In examining the assumptions for the linear regression model, we found that homoscedasticity and normality of errors did not appear to hold for some of the TCGA studies (Additional file 1: Figure S9, Additional file 1: Figure S10). Consequently, in addition to reporting *p*-values obtained from fitting linear regression models to each TCGA study, we also designed and applied a non-parametric randomization-based test for testing the association between predicted FCO and tumor/nontumor status and report the resulting p-values from this method as well. To obtain randomization-based p-values, we first constructed an empirical null distribution of test-statistics under the null hypothesis of no association between predicted FCO and tumor/nontumor status. Specifically, for each TCGA study, we randomly permuted tumor/nontumor status, fit a linear regression model adjusted for age, gender, race, and vital status (where available and relevant) with the permutated class label as an explanatory variable, and recorded the resulting test-statistic for the coefficient on tumor/nontumor status. This process was repeated 50,000 times within each TCGA study and used to obtain the empirical null distribution. Finally, we compared the observed test-statistic for the coefficient on tumor/nontumor status to the empirical null distribution of this statistic and computed the two-sided randomization-based *p*-value.

### Replication data sets

To replicate our findings, we used tumor and nontumor normal samples from 15 GEO data sets: (1) GSE49656 [32] contains 32 cholangiocarcinoma samples and 4 normal bile duct samples; (2) GSE53051 [33] contains 35 colon cancer samples and 18 normal colon samples, 9 lung cancer samples and 11 normal lung samples, 14 breast cancer samples and 10 normal breast samples, 29 pancreatic cancer samples and 12 normal pancreas samples, 70 thyroid cancer samples and 12 normal thyroid samples; (3) GSE52068 [34] contains 24 nasopharyngeal carcinoma and 24 normal nasopharyngeal epithelial samples; (4) GSE52826 [35] contains 4 esophageal squamous cell carcinoma samples, 4 paired adjacent normal surrounding tissues and 4 normal esophagus mucosa from healthy individuals; (5) GSE52955 [36] contains 17 renal tumor samples and 6 normal kidney samples, 25 bladder tumor samples and 5 normal bladder samples, 25 prostate tumor samples and 5 prostate normal samples; (6) GSE54503 [37] contains 66 hepatocellular carcinoma samples and 66 adjacent non-tumor tissue; (7) GSE56044 [38] contains 124 lung cancer samples 12 normal lung samples; (8) GSE75546 [39] contains 6 rectal cancer samples and 6 normal rectal samples; (9) GSE77871 [30] contains 18 adrenal cortical cancer samples and 6 normal adrenal samples; (10) GSE85845 [40] contains 8 lung cancer samples and 8 adjacent non-tumor samples; (11) GSE76938 [41] contains 73 prostate cancer samples and 63 normal prostate samples; (12) GSE112047 [42] contains 31 prostate cancer samples and 16 adjacent non-tumor samples; (13) GSE101961 [43] contains 121 normal breast samples; (14) GSE72245 [44] contains 118 breast cancer samples; (15) GSE106600 [45] contains 12 hematopoietic cell samples from patients with chronic phase chronic myeloid leukemia and 12 normal hematopoietic cell samples.

### Data processing and quality control

Level 3 Illumina Infinium HumanMethylation450 BeadChip array data on TCGA contains beta values calculated from background-corrected methylated (M) and unmethylated (U) array intensities as Beta = M/(M + U). In these data, probes having a common SNP within 10 bp of the interrogated CpG site or having overlaps with a repetitive element within 15 bp from the interrogated CpG site are masked as “NA” across all samples, as were probes with a non-detection probability (*P* > 0.01) in a given sample. Replication data sets, GSE52826 [32] and GSE54503 [34] contain average beta values processed by BeadStudio software; GSE49656 [29], GSE52955 [33] and GSE77871 [46] contain average beta values processed by the GenomeStudio software; GSE52068 [31], GSE75546 [36], GSE106600 [42] and GSE85845 [37] contain normalized average beta value processed by the GenomeStudio software; GSE56044 [35] and GSE72245 [41] contain peak-based normalized beta values; GSE53051 [33] and GSE112047 [39] contain normalized beta values by using the minfi package in Bioconductor; GSE101961 [40] contains normalized beta values by using the Subset-Quantile Within Array Normalization (SWAN); GSE76938 [38] contains normalized beta values using ComBat normalization. We previously evaluated the stability of the FCO estimations by excluding some of the 27 FCO markers using a leave-one-out combination, leave-two-out combination, until five probe combinations were removed. The results showed that though the potential error increases per probe removed, the estimates are stable in the absence of a small number of the probes [27]. For the purpose of quality control, we included only samples with at least 25 out of 27 CpGs in the FCO library. FCO was estimated in discovery data sets by using 25 CpGs in the FCO library due to quality control and in replication data sets, the full set of 27 CpGs constituting the FCO library was used.

### Sensitivity analyses for the decrease of FCO in tumor

As per the method of Qin et.al [47], we evaluated the tumor purity of tumor tissue samples on TCGA and examined the correlation between FCO and tumor purity. Furthermore, we used the TCGA tumor pathology tissue slide data on Biospecimen Core Resource (BCR) to examine the correlation between the percentage of leukocytes infiltration and the fractions of cells with FCO signature.

## Results

To describe the relative prevalence of fetal origin cells in human tumors compared with adjacent nontumor normal tissues, we applied our FCO signature to DNA methylation Infinium 450 K array data from TCGA. The analyses included 20 different tumor types studied by TCGA, and consisted of 6,795 primary tumor samples and 922 nontumor normal samples (Table 1).

**Table 1:** Baseline characteristics of TCGA tumor projects included in the study

TCGA Tumor Abbreviation	Tumor *n*	Nontumor normal *n*	Mean age (sd)	Male *n* (%)	White *n* (%)	Black *n* (%)	Asian *n* (%)	Other race *n* (%)
BLCA	418	21	68.60 (10.60)	319 (72.7)	351 (83.6)	25 (6.0)	44 (10.5)	0 (0.0)
BRCA	791	97	58.72 (13.34)	9 (1.0)	668 (76.6)	164 (18.8)	39 (4.5)	1 (0.1)
CESC	307	23	48.77 (13.79)	0 (0.0)	213 (77.7)	31 (11.3)	20 (7.3)	10(3.6)
CHOL	36	9	65.07 (12.46)	22 (48.9)	40 (88.9)	2 (4.4)	3 (6.7)	0 (0.0)
COAD	313	38	66.21 (13.21)	188 (53.9)	240 (75.7)	65 (20.5)	11 (3.5)	1 (0.3)
ESCA	185	16	63.41 (11.87)	168 (83.6)	130 (71.8)	5 (2.8)	46 (25.4)	0 (0.0)
GBM	140	140	60.44 (12.72)	81 (58.3)	107 (81.7)	24 (18.3)	0 (0.0)	0 (0.0)
HNSC	528	50	61.54 (11.82)	424 (73.4)	495 (88.1)	54 (9.6)	11 (2.0)	2 (0.4)
KIRC	324	160	62.54 (11.71)	316 (65.3)	421 (88.1)	55 (11.5)	2 (0.4)	0 (0.0)
LIHC	377	50	60.15 (13.79)	285 (66.7)	221 (53.4)	24 (5.8)	167 (40.3)	2 (0.5)
LUAD	473	32	65.37 (10.29)	236 (46.7)	392 (86.2)	57 (12.5)	6 (1.3)	0 (0.0)
LUSC	370	42	68.23 (8.85)	303 (73.5)	308 (90.3)	25 (7.3)	8 (2.3)	0 (0.0)
PAAD	184	10	65.46 (11.10)	108 (55.7)	170 (89.5)	8 (4.2)	12 (6.3)	0 (0.0)
PCPG	148	8	50.94 (3.12)	66 (44.0)	126 (86.3)	14 (9.6)	5 (3.4)	1 (0.7)
PRAD	502	50	61.64 (6.77)	552(100.0)	195 (94.2)	10 (4.8)	2 (1.0)	0 (0.0)
READ	98	7	63.57 (12.30)	56 (53.8)	76 (92.7)	5 (6.1)	1 (1.2)	0 (0.0)
SARC	261	4	61.52 (14.62)	120 (45.3)	232 (90.6)	18 (7.0)	6 (2.3)	0 (0.0)
STAD	395	63	65.78 (10.68)	259 (65.2)	255 (71.2)	13 (3.6)	89 (24.9)	1 (0.3)
THCA	507	56	47.64 (15.94)	150 (26.6)	372 (80.7)	33 (7.2)	55 (11.9)	1 (0.2)
UCEC	438	46	64.54 (11.19)	0 (0.0)	318 (72.1)	105 (23.8)	9 (2.0)	9 (2.0)
Total	6795	922	61.85 (13.60)	3730 (48.9)	5330 (80.4)	737 (11.1)	536 (8.1)	28 (0.4)

**Fig. 1:**
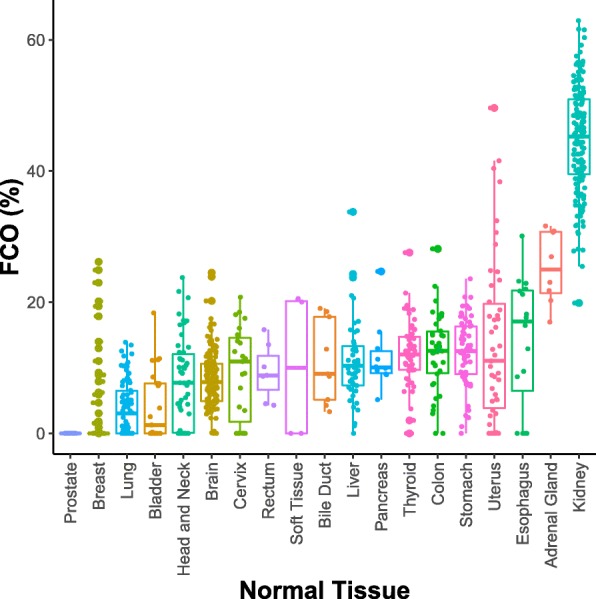
Distribution of predicted FCO (%) across different types of nontumor normal tissues

We first applied the FCO algorithm to nontumor normal tissue samples to infer the proportion of fetal origin cells across normal tissues. In our previous study, we showed the high FCO fraction in diverse fetal tissues and in sharp contrast, the minimal representation of the FCO signature in adult tissues [27]. Also, we demonstrated the high variability of the FCO across different types of fetal tissues and adult tissues respectively [27]. Consistent with our prior report [27], the fraction of fetal origin cells varied widely across different types of normal tissues. The mean FCO fraction varied from as low as 0% for prostate to as high as 44.9% for kidney (Fig. 1). We previously observed a global decrease of FCO cell fraction in blood leukocytes over the lifespan [27] and, therefore, we tested whether the inverse correlation between proportion of cells with the FCO signature and age would also exist in normal tissues. Across the 19 different types of normal tissues, there were six in which a significant inverse correlation between FCO and age was observed, and notable variation in the correlation across tissue types with correlation coefficients varying from − 1 for cervix to 0.037 for breast (Additional file 1: Figure S1).

**Fig. 2:**
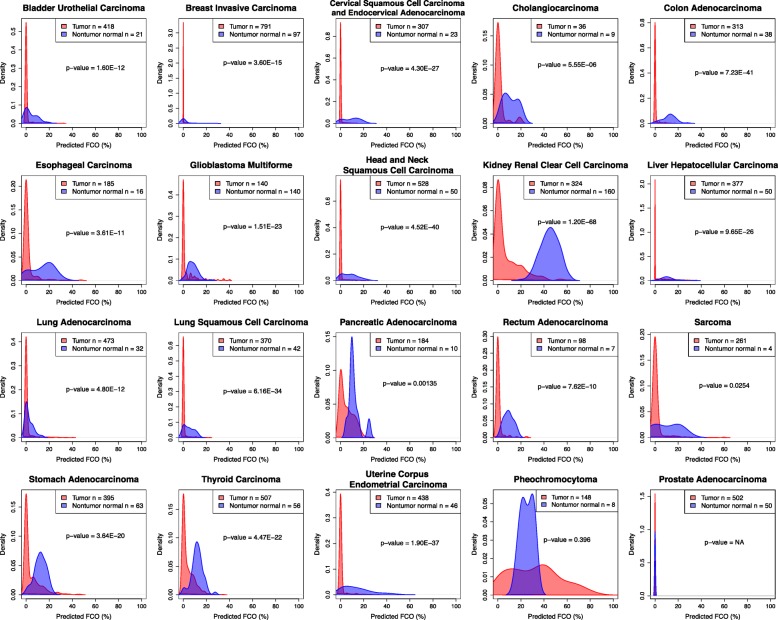
Kernel density plots of predicted FCO (%) in tumor and nontumor normal samples across different TCGA studies

Next, the FCO signal was estimated in tumor samples and compared with nontumor normal samples. Univariate analyses identified significantly lower proportions of cells with the FCO signature across all tumor types (*P* < 0.05), with the exception of prostate carcinoma and pheochromocytoma (Fig. 2). In prostate, the mean FCO was 0% in both normal tissue and tumor, and in pheochromocytoma, the FCO varied from 0 to 86%. We next tested the relationship of the FCO signature with tumor tissue status using linear models adjusted for potential confounders (e.g., age, gender, race and vital status) where possible, given the data available in the TCGA, and observed the same statistically significant differences of FCO between tumor and nontumor normal tissues (Table 2). To ensure that our results are robust to departure from model assumptions, we designed and applied a non-parametric randomization-based test which revealed little differences as compared to those obtained from the linear regression model, with 17/18 tumor types remaining statistically significant (Table 2). The one exception was sarcoma where randomization-based *p*-value was not significant, but approached significance, *p* = 0.061.

**Table 2:** P-values based on comparisons of the predicted FCO (%) between tumor and nontumor normal samples across different TCGA studies. *P*-values were obtained using a non-parametric Wilcoxon rank sum test, multiple linear regression model, and a non-parametric randomization-based testing procedure. P-values in PRAD are NA because FCO (%) in tumor and nontumor normal samples are both 0%

Tumor	Wilcoxon rank sum test	Linear regression	Randomization-based test
BLCA	1.60E-12	6.57E-08	0.00052
BRCA	3.60E-15	5.59E-22	<2E-05
CESC	4.30E-27	1.34E-51	<2E-05
CHOL	5.55E-06	8.63E-06	0.00016
COAD	7.23E-41	1.16E-61	<2E-05
ESCA	3.61E-11	2.21E-13	<2E-05
GBM	1.51E-23	7.94E-14	<2E-05
HNSC	4.52E-40	2.77E-58	<2E-05
KIRC	1.20E-68	9.05E-146	<2E-05
LIHC	9.65E-26	8.48E-17	<2E-05
LUAD	4.80E-12	0.00236	0.0216
LUSC	6.16E-34	2.78E-19	<2E-05
PAAD	0.00135	0.000307	0.00128
PRAD	NA	NA	NA
PCPG	0.396	3.84E-01	0.33
READ	7.62E-10	9.03E-09	0.00032
SARC	0.0254	0.00757	0.0607
STAD	3.64E-20	2.79E-13	<2E-05
THCA	4.47E-22	7.22E-26	<2E-05
UCEC	1.90E-37	2.32E-56	<2E-05

To investigate whether the decrease of FCO in tumor tissues is a result of leukocyte infiltration (which, in adults, have a very small FCO) [27, 48], we used direct estimates of leukocyte infiltration from TCGA. Where data were available, the correlation between the FCO signature proportion and proportion of infiltrating monocyte, lymphocyte, and neutrophils, for each tumor type indicated both that the FCO was not inversely correlated with any leukocyte infiltration in any tumor type and that the infiltration percentage was generally low (Additional file 1: Figure S2, Additional file 1: Figure S3, Additional file 1: Figure S4). In addition, we tested whether normal cell contamination of tumor tissue samples biased the proportion of cells with an FCO signature. We applied the InfiniumPurify function designed for estimating tumor purity based on DNA methylation Infinium 450 k array data to tumor tissue samples from TCGA [47]. The tumor purity varied across different tumor types (Additional file 1: Figure S5), and a significant inverse correlation between tumor purity and FCO was observed in nine tumor types, while the remaining showed little correlation (Additional file 1: Figure S6). The significant inverse correlations between FCO and tumor purity remained in eight tumor types after adjusting for age, gender, race and vital status, provided these data were available and relevant to adjust for (Additional file 1: Table S1). Although the FCO fraction decreases as tumor purity goes up in some tumor types, suggesting that normal cell contamination altered the FCO estimation in tumors to some extent, the significant drop of FCO in tumor compared to nontumor normal is still valid.

We next examined whether the FCO is associated with tumor stage and histological subtypes. Across 20 tumor projects in our study, eight (CHOL, GBM, KIRC, LIHC, PAAD, PCPG, STAD and THCA) have nonzero interquartile range (IQR) of FCO and thus were included in the analyses. Among these 8 tumor types, pheochromocytomas (PCPG) lacked tumor stage information and glioblastomas (GBM) by definition are all stage IV. Only kidney renal clear cell carcinoma (KIRC) of the remaining 6 tumor types showed a significant negative association between FCO and tumor stage (*P* = 3.79e-14, Additional file 1: Figure S7). Tumor histological subtype data was available for 4 (CHOL, GBM, PAAD, THCA) out of 8 tumor types with IQR of FCO larger than zero, however we found no statistically significant association between FCO and histological subtype among these tumors.

**Fig. 3:**
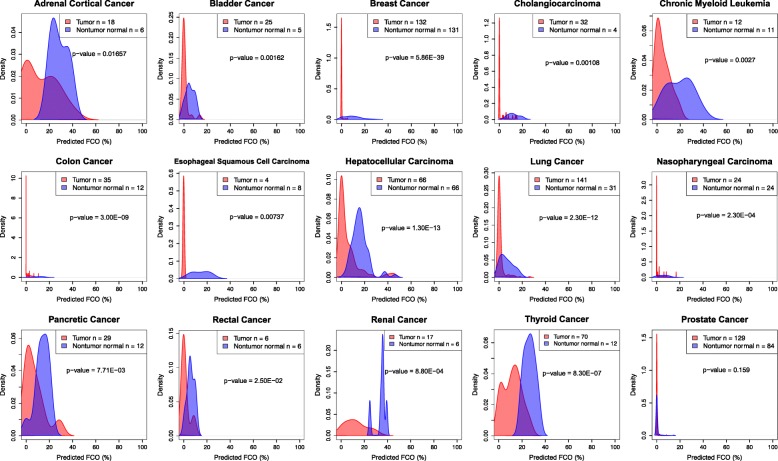
Kernel density plots of predicted FCO (%) in tumor and nontumor normal samples across different cancer types with available DNA methylation data in GEO

To replicate our findings, we accessed multiple independent data sets deposited in Gene Expression Omnibus (GEO) that included DNA methylation Infinium 450 K array measurements on tumor and nontumor normal tissues. Specifically, we applied our approach to infer the proportion of cells with the FCO signature in 15 GEO data sets, including 15 different tumor types, which comprised 740 primary tumor tissue samples and 424 normal tissue samples (Table 3). These data confirmed our previous results in that among the 15 tumor types forming our replication data, a significantly lower FCO was observed in tumor versus normal tissue in 14 of the 15 tumor types (Table 3, Fig. 3). Consistent with our TCGA analysis, FCO in prostate tumors was indistinguishable from normal tissue.

**Table 3:** Comparisons of the predicted FCO (%) between tumor and nontumor normal samples from GEO replication data sets

Cancer	Tumor n	Nontumor normal n	Wilcoxon rank sum test *p*-values	Mean age (sd)	Male n	Female n	Data source
Adrenal Cortical Cancer	18	6	0.01657	NA	NA	NA	GSE77871
Bladder Cancer	25	5	0.00162	NA	NA	NA	GSE52955
Breast Cancer	132	131	5.86E-39	47.31 (14.74)	0	252	GSE53051, GSE72245, GSE101961
Cholangiocarcinoma	32	4	0.00108	NA	NA	NA	GSE49656
Chronic Myeloid Leukemia	12	11	0.0027	NA	NA	NA	GSE106600
Colon Cancer	35	18	3.00E-09	67.47 (11.26)	5	2	GSE53051
Esophageal Squamous Cell Carcinoma	4	8	0.00737	NA	8	4	GSE52826
Hepatocellular Carcinoma	66	66	1.30E-13	NA	100	32	GSE54503
Lung Cancer	141	31	2.30E-12	63.50 (8.50)	8	2	GSE53051, GSE56044
Nasopharyngeal Carcinoma	24	24	2.30E-04	42.96 (10.28)	30	18	GSE52068
Pancreatic Cancer	29	12	7.71E-03	63.20 (16.24)	7	3	GSE53051
Prostate Cancer	129	84	0.159	60.21 (7.77)	213	0	GSE52955, GSE76938, GSE112047
Renal Cancer	17	6	8.80E-04	NA	NA	NA	GSE52955
Rectal Cancer	6	6	2.50E-02	65.50 (8.80)	4	8	GSE75546
Thyroid Cancer	70	12	8.30E-07	48.35 (14.90)	21	61	GSE53051
Total	740	424

Finally, since cancer stem cells share properties and surface markers with embryonic stem cells [18] we sought to directly examine their FCO. We applied the FCO algorithm to GEO data sets GSE80241 [49], representing 6 pancreatic ductal adenocarcinoma stem cell samples, and GSE92462 [50], including 22 glioma stem cell samples. FCO estimates were zero in both pancreatic ductal adenocarcinoma stem cells and in all but one glioma stem cell sample (Additional file 1: Table S2). Further, among 27 FCO CpGs, 3 (cg10338787, cg17310258 and cg16154155) are associated with EZH2. We plotted the methylation beta values of these three loci in pancreatic carcinoma samples, normal pancreatic tissue samples and pancreatic cancer stem cell samples from GEO data sets GSE53051 [33] and GSE80241 [49]. We examined methylation proportions in 29 pancreatic carcinoma samples, 12 normal pancreatic tissue samples and 6 pancreatic cancer stem cell samples. The profiles of EZH2 related CpGs in pancreatic cancer stem cells are distinguished from pancreatic tumor and normal samples as those loci are largely methylated in pancreatic cancer stem cells (Additional file 1: Figure S8).

## Discussion

We observed significant variation in the FCO signature in multiple normal tissues, consistent with our prior work [27]. Since the FCO signature was designed to reflect the proportion of cells that are of fetal origin [27], this suggests that normal tissues vary with respect to their cellular components that retain embryonic lineage. One example of this that could explain the relatively elevated FCO in normal kidney is the known large proportion of tissue-resident macrophages found in the kidney [51, 52]. These macrophages are embryonically-derived and would therefore be excellent candidates for having a high FCO. If this were the case, the elevated FCO in this constituent component of the kidney would drive the normal tissue signal to be elevated. In addition, the mechanism(s) responsible for the inverse correlation between FCO and age in multiple tissues remains unclear. It might arise as a result of the selective loss of constituent cells that are of embryonic lineage, such as the resident macrophages [53]. The FCO fraction varied from as low as 0% for prostate to as high as 44.9% for kidney is of interest; we posit that cells that retain the FCO signature might contribute to repair and regeneration in a given tissue. A further understanding of this awaits direct investigation of the FCO of the individual cellular components of normal tissues.

Though the types of cells that specifically account for the fetal origin signal remain unclear, there are several possible explanations for our findings in tumors themselves; it could be that most cancer cells are free of any FCO signal and that the rapid proliferation of cancer cells replaces the normal cells that are of fetal origin (with a higher FCO signal). This conforms with the prominent paradigm for explaining tumor heterogeneity – the hierarchical cancer stem cell model. The cancer stem cells acquire pluripotency during carcinogenesis. As a result, it seems likely that only a small number of cancer cells would retain any embryonic-like state and thus, have a high FCO. As those embryonic-like cancer cells differentiate and proliferate, the FCO signal might decrease in the progeny cells. The origin of cancer stem cells is not well established, but it is hypothesized that the cancer stem cells can arise from adult stem or progenitor cells, or possibly, the dedifferentiation of mature somatic cells [17]. Regardless of their origin, the dedifferentiation process that gives rise to the cancer stem cells could generate cells with a high FCO signal that is not retained in their progeny cancer cells. In this scenario, the low FCO signal in tumor samples indicates the rarity of cancer stem cells. While this remains a formal possibility, the limited data analyzed here suggest that cancer stem cells do not have consistently high FCO signals, making this scenario less plausible.

Cancer proliferation models proposed over several decades include the hierarchical cancer stem cell model and the stochastic clonal evolution model [54]. The former model is supported by recent research indicating that heterogeneous tumor cells develop over time as cancer stem cells differentiate via genetic and epigenetic alterations [55–58]. As the FCO signature is contained at a high level in induced pluripotent stem cells [27], the embryonic-like character of cancer stem cells and the striking similarities between tumor development and the generation of induced pluripotent stem cells might suggest that tumors would display an increase in the FCO signal. However, our findings are at odds with this; we found a decrease in the FCO arises in almost all tumors that cannot be explained by either leukocyte invasion or normal tissue contamination, and we observed a very low FCO signal in pancreatic ductal adenocarcinoma stem cells and glioma stem cells. This would perhaps suggest that cancer stem cells do not employ the normal embryonic lineage pathways in the process of malignant degeneration.

Further, our observation of a diminished FCO in tumors is seemingly at odds with reports that DNA hypermethylation in cancer preferentially targets the subset of polycomb repressor loci in cancer stem cells that are developmental regulators [59]. This seeming contradiction might suggest that either the cancer stem cells are quite rare in any tumor and that the cancer stem cell progeny quickly lose methylation or that the cancer stem cells differ in their driver gene content by tissue such that our library would not capture their character (as they are not invariant).

The major cancer stem cell specific pathways, including phosphatidylinositol 3-kinase (*PI3K*)/Akt/mammalian target of rapamycin (*mTOR*), maternal embryonic leucine zipper kinase (*MELK*), *NOTCH1*, and Wnt/β-catenin, and genes (including *CD133, CD24*, *CD44*, *OCT4*, *SOX2*, *NANOG* and *ALDH1A1*), maintain cancer stem cell properties [60]. However, the major genes and pathways identified in FCO signature [27] do not have substantial overlaps with these pathways. The FCO genes and pathways are primarily related to embryonic development and embryonic stem cell epigenetic marks and these are distinct from those driving cancer features, such as: tumor progression, apoptosis resistance, chemo- and radiotherapy resistance and tumor recurrence. The single gene identified as overrepresented in both FCO signature loci and cancer stem cell is *EZH2*. *EZH2* is a component of the polycomb repressor complex, which is responsible for maintaining stemness, and it has also been reported to be involved in the genesis of numerous malignancies [46, 61]. Thus, its role in both embryogenesis and cancer may be somewhat unique.

Another observation we found interesting is the large range and variation of FCO in pheochromocytoma. The FCO fraction in pheochromocytoma varied from 0 to 86% and the significant difference of FCO between tumor tissue and nontumor normal tissue we observed in other cancer types didn’t hold true for pheochromocytoma. One possible explanation for that is the origin of tumor cells differs in different tumor subtypes. Pheochromocytoma is derived from chromaffin cells of the adrenal medulla [62]. Perhaps the large variation of FCO in pheochrocytoma is attributed to the differences in the proportion of FCO cells in adrenal medulla vs the cortex. In addition, we observed that adrenal cortical tumor, which has a low fraction of FCO, is a more common tumor subtype than pheochromocytoma, which is a medullary tumor and has a large range and variation of FCO. Further investigations on how FCO distribution in an organ is related to the process of carcinogenesis are needed.

The FCO signature is designed to trace fetal origin cells; the CpGs included in the FCO signature library are putatively inherited from embryonic stem cells [27]. Given the observation that the FCO signal is low in cancer stem cells and majority of tumor cells, one possible explanation is that tumors only arise from cells not carrying the FCO signature; an alternative would be that tumors could arise from cells with FCO signature and the FCO change during carcinogenesis is attributed to the amount of FCO cells presented in the original site of the malignancy or the FCO signature is unstable during the process of carcinogenesis and thus lost. In sum, our findings suggest that tumors contain a relatively small fraction of cells of embryonic lineage if the FCO signature is stable during the malignant degeneration of a cell, at least from the perspective of DNA methylation.

While our results point to a significant absence of FCO in tumor tissues, we recognize some limitations. The major body of cancer tissue and normal tissue we analyzed came from TCGA and were based on the Infinium HumanMethylation450K BeadChip array. Our FCO deconvolution algorithm used a library of 27 CpGs that represents a phenotypic block of differentially methylated regions for estimating the proportion of cells in a mixture of cells that are of fetal origin. Among 27 CpGs in the FCO library, two were removed in TCGA methylation data. As a result, we used 25 CpGs in the library to do the FCO estimation. We previously demonstrated that the alteration of FCO estimation is minimal in the absence of a small number of probes in the FCO library [27]. Furthermore, the GEO data, which contains the full set of 27 CpGs, were used to validate the absence of FCO signal in tumor tissue.

Another limitation of our study is the mixed normalization protocols used in the data. The FCO algorithm was developed based on DNA methylation beta values normalized by the Funnorm function in minfi Bioconductor package. Consequently, the most appropriate normalization protocol to apply to DNA methylation array data in order to be consistent with FCO algorithm is Funnorm. However, the Level 3 TCGA used in this study did not include such normalization. While the methylation data on TCGA are raw average beta values, the normalization protocols applied on methylation data retrieved from GEO varied across studies. In spite of this, we believe that the differing normalization protocols had a minimal effect on FCO estimation as we have showed the reliability of the algorithm by applying it to multiple different GEO data sets regardless of the normalization protocol in our FCO development paper [27]. Also, the same approach was applied to tumor and nontumor specimens, which would limit normalization-based biases from impacting our results.

Finally, the limited numbers for some of the tumor types examined could lead to bias. We have attempted to mitigate this problem by adding additional analysis of publically available data sets, where possible.

## Conclusions

Future studies are needed to interrogate the specific types of cells that show a high FCO signal. The variation in FCO across different types of normal tissues likely reflects the underlying cellular composition of these tissues. Aging may change the FCO as a result of selective loss of cells of embryonic lineage. The process of carcinogenesis essentially universally diminishes the FCO; the precise mechanism(s) responsible for this are unclear but our data suggest that cancer development itself is substantially devoid of recapitulation of normal embryologic processes.

## Additional files

Additional file 1: **Figure S1** Correlations between age and fraction of cells with FCO signal in different types of normal tissues on TCGA. **Figure S2** Correlations between monocyte infiltration percentage and fraction of cells with FCO signal in different types of tumors on TCGA. **Figure S3** Correlations between lymphocyte infiltration percentage and fraction of cells with FCO signal in different types of tumors on TCGA. **Figure S4** Correlations between neutrophils infiltration percentage and fraction of cells with FCO signal in different types of tumors on TCGA. **Figure S5** The distribution of tumor purity across different types of tumors on TCGA. **Figure S6** Correlations between tumor purity and fraction of cells with FCO signal in different types of tumors on TCGA. **Figure S7** The FCO signal decreases as tumor stage increases in kidney renal clear cell carcinoma. **Figure S8** Methylation status of EZH2 related CpGs from FCO library in normal pancreatic tissue, pancreatic carcinoma and pancreatic carcinoma stem cell. **Figure S9** Normal QQ-plots showing the distribution of residuals from linear regression fits in TCGA tumor projects. **Figure S10** Spread-Location plots showing the spread of residuals along the ranges of predictors from linear regression fits in TCGA tumor projects. **Table S1** *P*-values based on comparisons of the predicted FCO (%) and tumor purity after adjusting for age, gender, race and vital status using multiple linear regression models across different TCGA studies. **Table S2** FCO in pancreatic ductal adenocarcinoma stem cells from GEO data set GSE80241 and glioma stem cells from GEO data set GSE92462. (DOCX 2395 kb)

## Data Availability

The datasets analyzed during the current study are available on The Cancer Genome Atlas (TCGA) https://portal.gdc.cancer.gov and the Gene Expression Omnibus data repository https://www.ncbi.nlm.nih.gov/geo/ (Accession numbers: GSE49656, GSE53051, GSE52068, GSE52826, GSE52955, GSE54503, GSE56044, GSE75546, GSE77871, GSE85845, GSE76938, GSE112047, GSE101961, GSE72245, GSE106600, GSE80241, GSE92462).

## Abbreviations

BLCA: bladder urothelial carcinoma
BRCA: breast invasive carcinoma
CESC: cervical squamous cell carcinoma and endocervical adenocarcinoma
CHOL: cholangiocarcinoma
COAD: colon adenocarcinoma
ESC: embryonic stem cell
ESCA: esophageal carcinoma
FCO: fetal cell origin
GBM: glioblastoma multiforme
HNSC: head and neck squamous cell carcinoma
IQR: interquartile range
KIRC: kidney renal clear cell carcinoma
LIHC: liver hepatocellular carcinoma
LUAD: lung adenocarcinoma
LUSC: lung squamous cell carcinoma
PAAD: pancreatic adenocarcinoma
PCPG: pheochromocytoma and paraganglioma
PRAD: prostate adenocarcinoma
READ: rectum adenocarcinoma
SARC: sarcoma
STAD: stomach adenocarcinoma
TCGA: The Cancer Genome Atlas
THCA: thyroid carcinoma
THYM: thymoma
UCEC: uterine corpus endometrial carcinoma
