# Supplementary material for: Absence of an embryonic stem cell DNA methylation signature in human cancer
Source: BMC Cancer. 2019 Jul 19;19:711. doi: 10.1186/s12885-019-5932-6 (PMC6642562; doi:10.1186/s12885-019-5932-6)
Supplement: Supplementary file 1 — Figure S1 Correlations between age and fraction of cells with FCO signal in different types of normal tissues on TCGA. Figure S2 Correlations between monocyte infiltration percentage and fraction of cells with FCO signal in different types of tumors on TCGA. Figure S3 Correlations between lymphocyte infiltration percentage and fraction of cells with FCO signal in different types of tumors on TCGA. Figure S4 Correlations between neutrophils infiltration percentage and fraction of cells with FCO signal in different types of tumors on TCGA. Figure S5 The distribution of tumor purity across different types of tumors on TCGA. Figure S6 Correlations between tumor purity and fraction of cells with FCO signal in different types of tumors on TCGA. Figure S7 The FCO signal decreases as tumor stage increases in kidney renal clear cell carcinoma. Figure S8 Methylation status of EZH2 related CpGs from FCO library in normal pancreatic tissue, pancreatic carcinoma and pancreatic carcinoma stem cell. Figure S9 Normal QQ-plots showing the distribution of residuals from linear regression fits in TCGA tumor projects. Figure S10 Spread-Location plots showing the spread of residuals along the ranges of predictors from linear regression fits in TCGA tumor projects. Table S1 P-values based on comparisons of the predicted FCO (%) and tumor purity after adjusting for age, gender, race and vital status using multiple linear regression models across different TCGA studies. Table S2 FCO in pancreatic ductal adenocarcinoma stem cells from GEO data set GSE80241 and glioma stem cells from GEO data set GSE92462. (DOCX 2395 kb) [file 12885_2019_5932_MOESM1_ESM.docx]

**
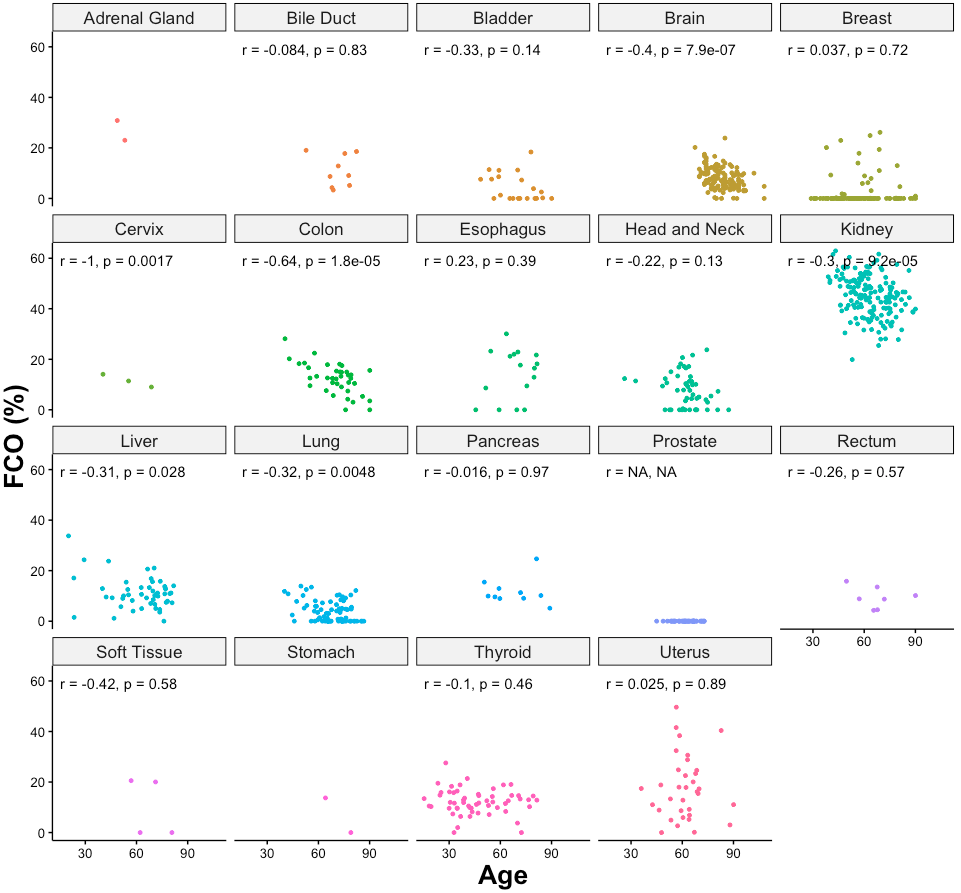
**

**Figure S1.** Correlations between age and fraction of cells with FCO signal in different types of normal tissues on TCGA.


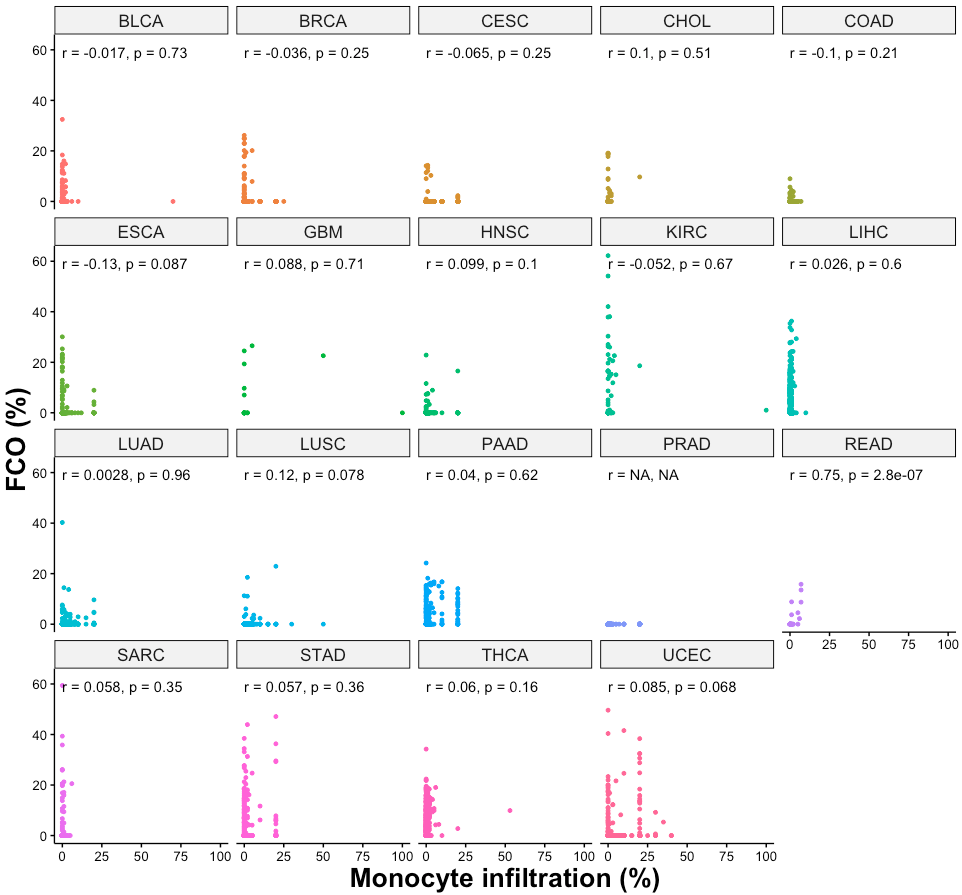


**Figure S2.** Correlations between monocyte infiltration percentage and fraction of cells with FCO signal in different types of tumors on TCGA.


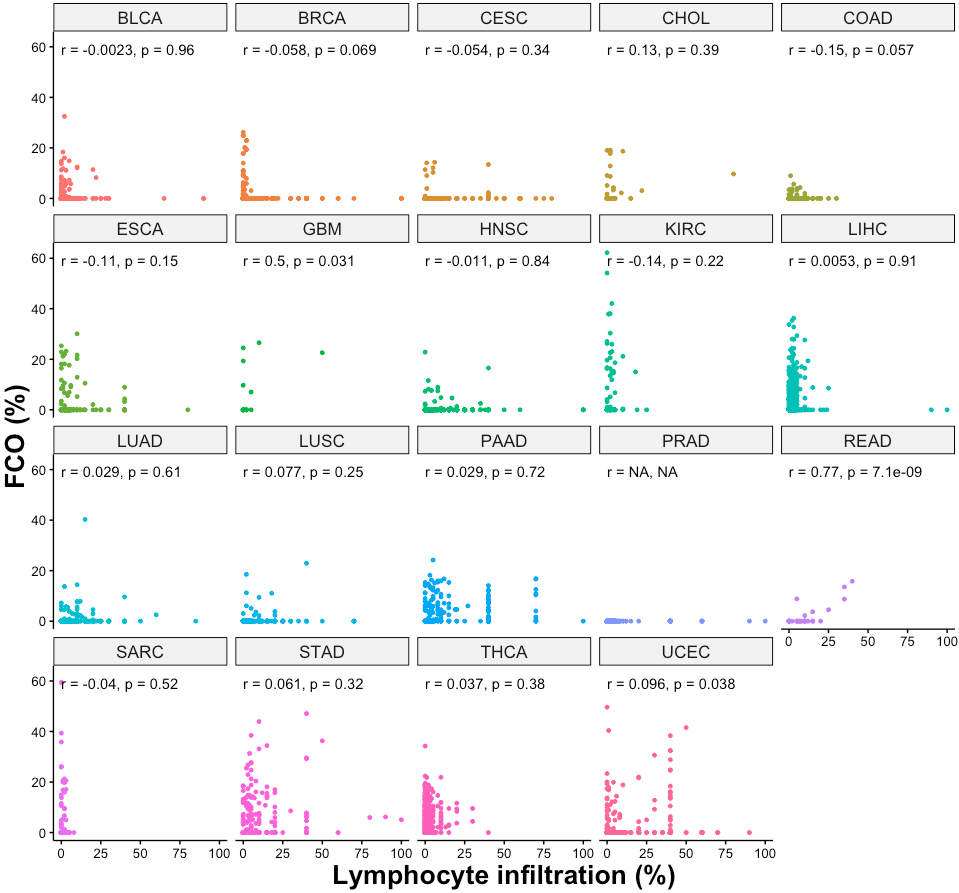


**Figure S3.** Correlations between lymphocyte infiltration percentage and fraction of cells with FCO signal in different types of tumors on TCGA.


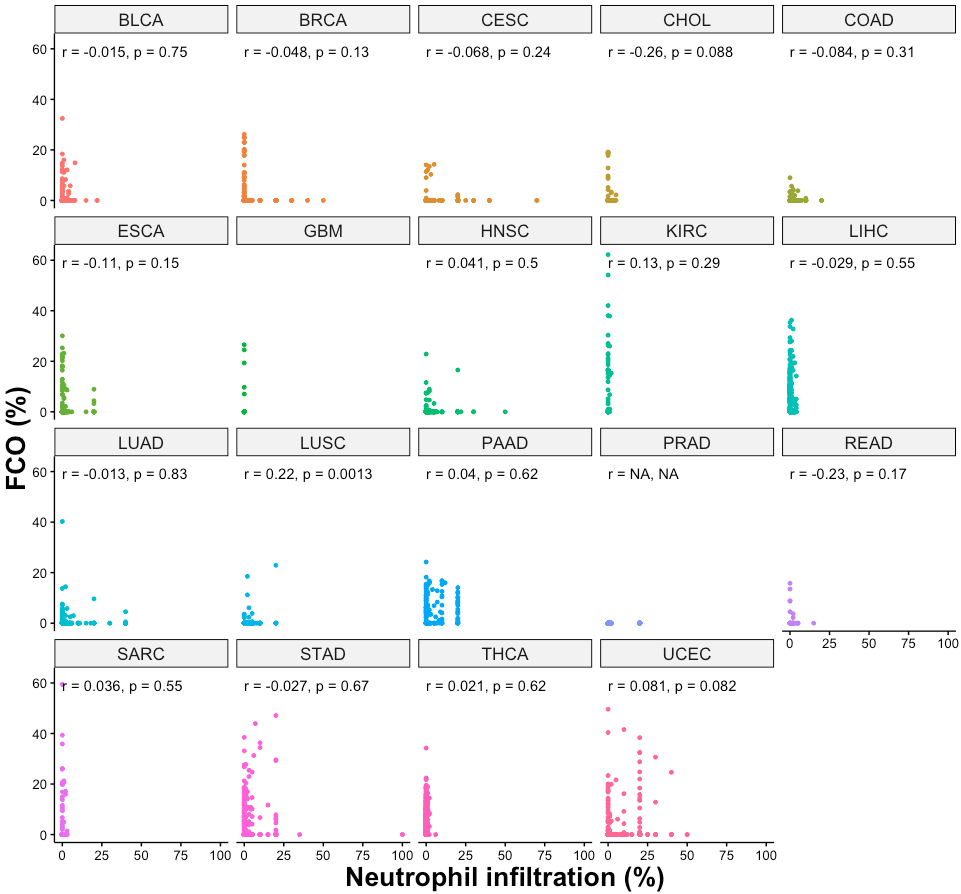


**Figure S4.** Correlations between neutrophils infiltration percentage and fraction of cells with FCO signal in different types of tumors on TCGA.


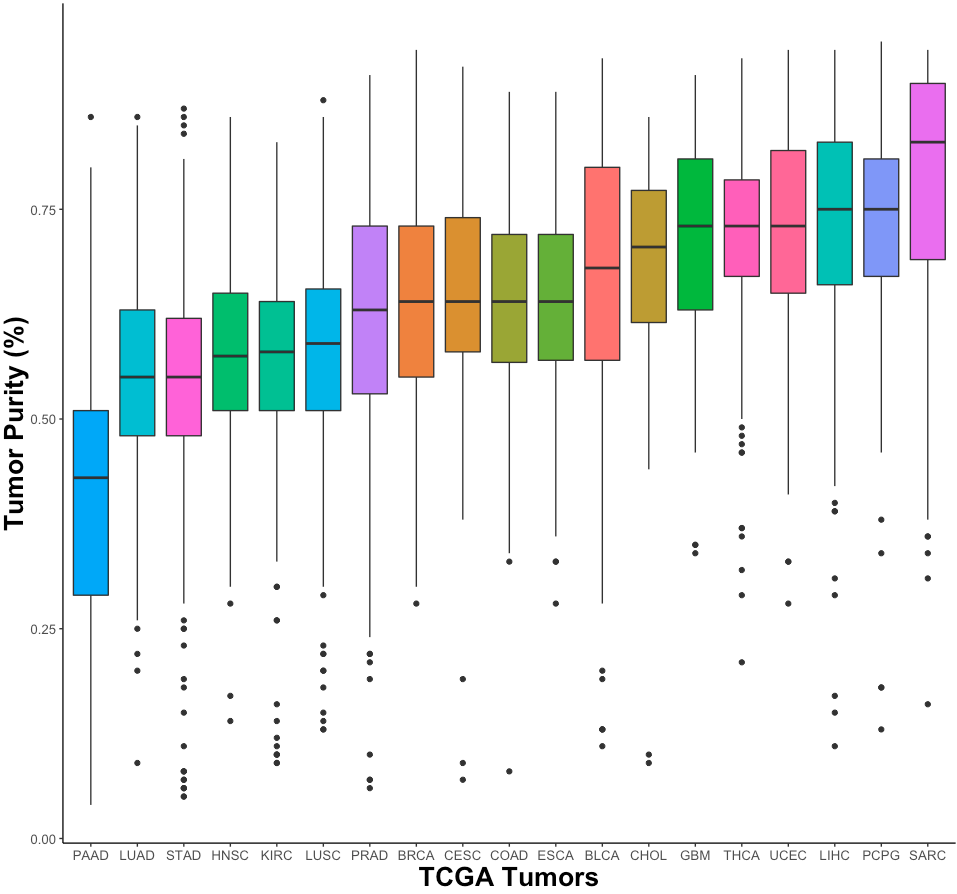


**Figure S5.** The distribution of tumor purity across different types of tumors on TCGA.


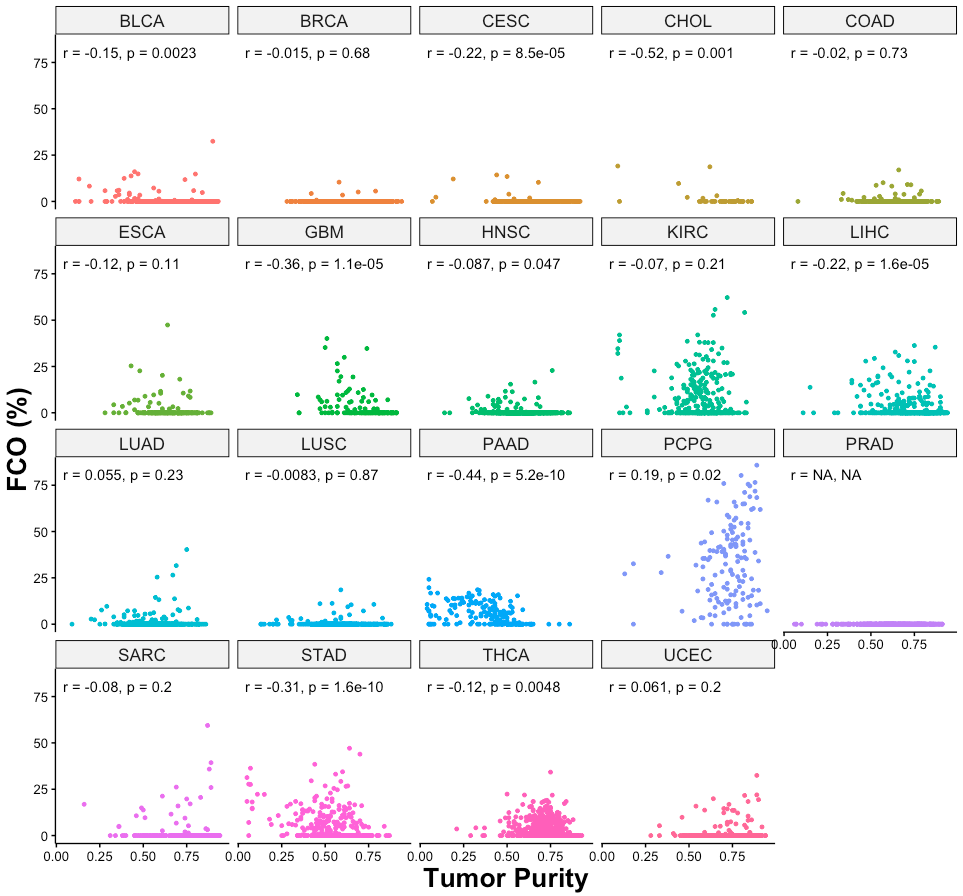


**Figure S6.** Correlations between tumor purity and fraction of cells with FCO signal in different types of tumors on TCGA.

**Figure S7.** The FCO signal decreases as tumor stage increases in kidney renal clear cell carcinoma.


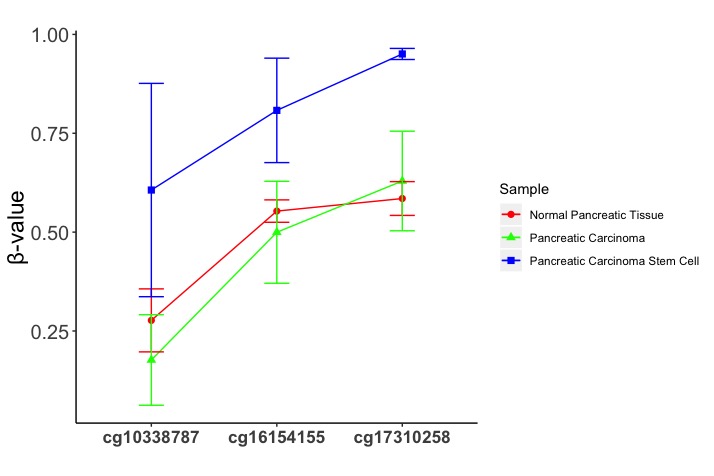


**Figure S8.** Methylation status of EZH2 related CpGs from FCO library in normal pancreatic tissue, pancreatic carcinoma and pancreatic carcinoma stem cell.

**Figure S9.** Normal QQ-plots showing the distribution of residuals from linear regression fits in TCGA tumor projects.

**Figure S10,** Spread-Location plots showing the spread of residuals along the ranges of predictors from linear regression fits in TCGA tumor projects.

| **Tumor** | **Linear Regression P-value** |
| --- | --- |
| BLCA | 0.00534 |
| BRCA | 0.244 |
| CESC | 0.000191 |
| CHOL | 0.0139 |
| COAD | 0.894 |
| ESCA | 0.1 |
| GBM | 3.53E-05 |
| HNSC | 0.0651 |
| KIRC | 0.111 |
| LIHC | 6.10E-05 |
| LUAD | 0.23 |
| LUSC | 0.771 |
| PAAD | 1.74E-09 |
| PRAD | NA |
| PCPG | 0.0095 |
| SARC | 0.147 |
| STAD | 6.85E-09 |
| THCA | 0.0116 |
| UCEC | 0.22 |

**Table S1.** P-values based on comparisons of the predicted FCO (%) and tumor purity after adjusting for age, gender, race and vital status using multiple linear regression models across different TCGA studies.

| Pancreatic Ductal Adenocarcinoma Stem Cells | |
| --- | --- |
| ID | FCO(%) |
| GSM2122595 | 0 |
| GSM2122597 | 0 |
| GSM2122599 | 0 |
| GSM2122601 | 0 |
| GSM2122603 | 0 |
| GSM2122605 | 0 |
|  |  |
| Glioma Stem Cells |  |
| ID | FCO(%) |
| GSM2430095 | 0 |
| GSM2430096 | 0 |
| GSM2430097 | 0 |
| GSM2430098 | 0 |
| GSM2430099 | 0 |
| GSM2430100 | 0 |
| GSM2430101 | 0 |
| GSM2430102 | 0 |
| GSM2430103 | 0 |
| GSM2430105 | 0 |
| GSM2430106 | 0 |
| GSM2430109 | 0 |
| GSM2430111 | 0 |
| GSM2430113 | 0 |
| GSM2430115 | 0 |
| GSM2430117 | 0 |
| GSM2430119 | 4.85 |
| GSM2430122 | 0 |
| GSM2430124 | 0 |
| GSM2430127 | 0 |
| GSM2430129 | 0 |
| GSM2430131 | 0 |

**Table S2.** FCO in pancreatic ductal adenocarcinoma stem cells from GEO data set GSE80241 and glioma stem cells from GEO data set GSE92462.
